# A Naturally Occurring Antibody Fragment Neutralizes Infectivity of Diverse Infectious Agents

**DOI:** 10.1038/srep35018

**Published:** 2016-10-11

**Authors:** Luciano Polonelli, Tecla Ciociola, Lisa Elviri, Pier Paolo Zanello, Laura Giovati, Denise C. Arruda, Julián E. Muñoz, Renato A. Mortara, Giulia Morace, Elisa Borghi, Serena Galati, Oriano Marin, Claudio Casoli, Elisabetta Pilotti, Paola Ronzi, Luiz R. Travassos, Walter Magliani, Stefania Conti

**Affiliations:** 1 Microbiology and Virology Unit, Department of Biomedical, Biotechnological, and Translational Sciences, Università degli Studi di Parma, Parma, Italy; 2 Department of Pharmacy, Università degli Studi di Parma, Parma, Italy; 3 Experimental Oncology Unit, Federal University of São Paulo (UNIFESP), São Paulo, Brazil; 4 Department of Microbiology, University of São Paulo, São Paulo, Brazil; 5 Department of Microbiology, Immunology and Parasitology, UNIFESP, São Paulo, Brazil; 6 Department of Health Sciences, Università degli Studi di Milano, Milan, Italy; 7 Department of Life Sciences, Università degli Studi di Parma, Parma, Italy; 8 Department of Biomedical Sciences, Università degli Studi di Padova, Padua, Italy; 9 “L. Sacco” Department of Biomedical and Clinical Sciences, Università degli Studi di Milano, Milan, Italy; 10 GEMIB Laboratory, Centre for Medical Research and Molecular Diagnostic, Parma, Italy

## Abstract

A phosphorylated peptide, named K40H, derived from the constant region of IgMs was detected in human serum by liquid chromatography coupled to high-resolution mass spectrometry. Synthetic K40H proved to exert a potent *in vitro* activity against fungal pathogens, and to inhibit HIV-1 replication *in vitro* and *ex vivo*. It also showed a therapeutic effect against an experimental infection by *Candida albicans* in the invertebrate model *Galleria mellonella*. K40H represents the proof of concept of the innate role that naturally occurring antibody fragments may exert against infectious agents, shedding a new light upon the posthumous role of antibodies and opening a new scenario on the multifaceted functionality of humoral immunity.

Previous studies have demonstrated that synthetic peptides, representative of the sequences of complementarity determining regions (CDRs) of antibodies (Abs), could exert *in vitro*, *ex vivo* and/or *in vivo* antimicrobial, antiviral, antitumour and/or immunomodulatory activities, plausibly mediated by different mechanisms of action and regardless of the specificity of the Abs they belong to^1^,^2^. On the basis of these results and in consideration of the possibility of peptide engineering and chemical optimisation, associated with new delivery mechanisms, it has been postulated that Ab internal peptide sequences could represent an unlimited source of therapeutic agents against infectious and neoplastic diseases^3^. The unlikelihood, however, that significant amounts of CDR fragments occur *in vivo*, in physiological conditions, could minimise their role as supplementary immune factors.

Synthetic peptides representing fragments of the constant regions of the major classes of immunoglobulins (IgG, IgM, IgA) (Fc-peptides), which were selected according to different criteria, including potential cleavage sites by trypsin- and/or chymotrypsin-like proteases, and cleavage probability, were also shown to exert, *in vitro* and/or *in vivo*, antimicrobial and/or immunomodulatory activities^4^,^5^. Peptides derived from single amino acid substitutions of bioactive CDR-derived and Fc-peptides proved to display further differential biological activities^1^,^4^. These findings led us to speculate that physiological Ab fragments could play a posthumous role in natural immunity.

In the present work, a liquid chromatography-electrospray ionisation-high resolution multistage mass spectrometry (LC-ESI-HRMS/MS) method was applied for the peptide profiling of serum samples, aiming at the identification of Ab-fragments. The previously described Fc-peptides N10K and T11F^4^ were not found in sera from nine healthy individuals within the detection limits of the adopted method. In contrast, a phosphorylated peptide (K40H), derived from the constant region of human IgMs, was detected in the human serum. As proof of concept of the anti-infective potential of Ab fragments in the serum, we observed that peptide K40H was able to exert *in vitro* and *ex vivo* antiviral activity against HIV-1 and *in vitro* fungicidal activity against *Candida albicans*, including strains resistant to conventional antifungal drugs, *Cryptococcus neoformans* and *Malassezia furfur*. Significantly, K40H also showed a therapeutic effect against experimental candidiasis in an invertebrate host model (*Galleria mellonella*), without exerting toxic or genotoxic effects against mammalian cells.

## Results

### Characterisation of serum Ab peptide K40H

As a first step, nine individual human serum samples, purified as reported in the Methods section, were analysed by LC-ESI-HRMS/MS with the aim to identify the Fc-peptides N10K and T11F. The spike of reference standards of the selected peptides in serum samples allowed for estimating the detection limit of the LC-HRMS method at the concentration range of 7–10 nM. Although there could have been detectable amounts of Fc-peptides in physiological conditions, none were found in the analysed sera.

A wide search for other possible Ab-derived peptides was then pursued. Peptides were searched in human sera, after development of a suitable LC-ESI-HRMS/MS method, through Data Dependent Acquisition mode, to obtain a global peptide profile overview. MS/MS data were initially processed against the whole UniProt human protein database. Specificity and selectivity of the adopted technique allowed for the identification of several peptides in human sera. The results were similar to those observed in other studies on human sera^6^. Among the detected peptides, a fragment of complement component 3c (SEETKENEGFTVTAEGK, S17K) was synthesised and used as a control in selected experiments.

In order to detect Ab-derived peptides, MS/MS data were further processed against a customised database, in which all-possible human Ig sequence information was collected. Every MS/MS spectrum with a match against this database was then re-searched against the collection of all human protein sequences to avoid false positive results. Under these search conditions, few possible Ab-derived peptides were detected with acceptable database match score. Among these, a fragment of IgM constant region conserved domain (Supplementary Fig. 1), with sequence KSTKLTCLVTDLTTYDSVpTIpSWTRQNGEAVKTHTNISESH, named K40H, was selected as an example of Ab-derived peptide for further studies. When processing all the nine serum samples against the selected sequence, two peptides with the same sequence but different amino acid length (39 residues) and post-translational modifications (40 residues and one phosphorylation site), were detected in 2 other serum samples. The actual concentration of K40H peptide found in human serum is not known, since only a qualitative analysis was performed.

### *In vitro* biological activity of peptide K40H

#### Fungicidal activity

**Figure 1:**
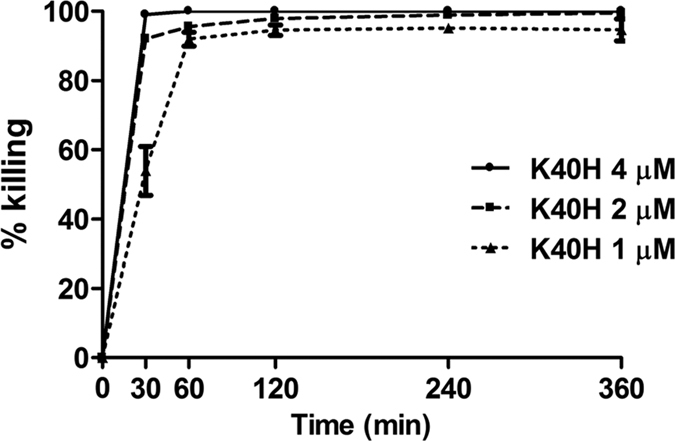
Time kinetics of K40H killing of *Candida albicans*. Viable yeast cells were incubated up to 6 h in the absence (control) or presence of K40H at different concentrations. At different times (30, 60, 120, 240, and 360 min), the yeast suspensions were plated on Sabouraud dextrose agar, and colony forming units were enumerated after 48 h. Percentual killing was calculated with reference to the number of colonies in controls. Results are representative of at least 2 independent experiments. Each experiment was performed in triplicate.

Peptide K40H exhibited a significant microbicidal effect against all the investigated fungal strains, with half maximal effective concentration (EC_50_) values ranging from 0.64 to 2.74 μM (Table 1). Time-killing curves, determined by incubation of *C. albicans* SC5314 cells with K40H at three different concentrations, demonstrated a rapid candidacidal effect of the peptide. In particular, nearly 100% and more than 90% killing was observed within 30 min at the highest concentrations tested (4 and 2 μM, respectively). At the lowest peptide concentration (1 μM) less than 50% of the yeast cells were viable after 30 min of incubation and less than 10% after 1 h (Fig. 1).

The irrelevant peptide S17K showed no candidacidal activity (0% killing) even at the highest tested concentration (40 μM, not shown in figure).

#### Antiviral activity

**Figure 2:**
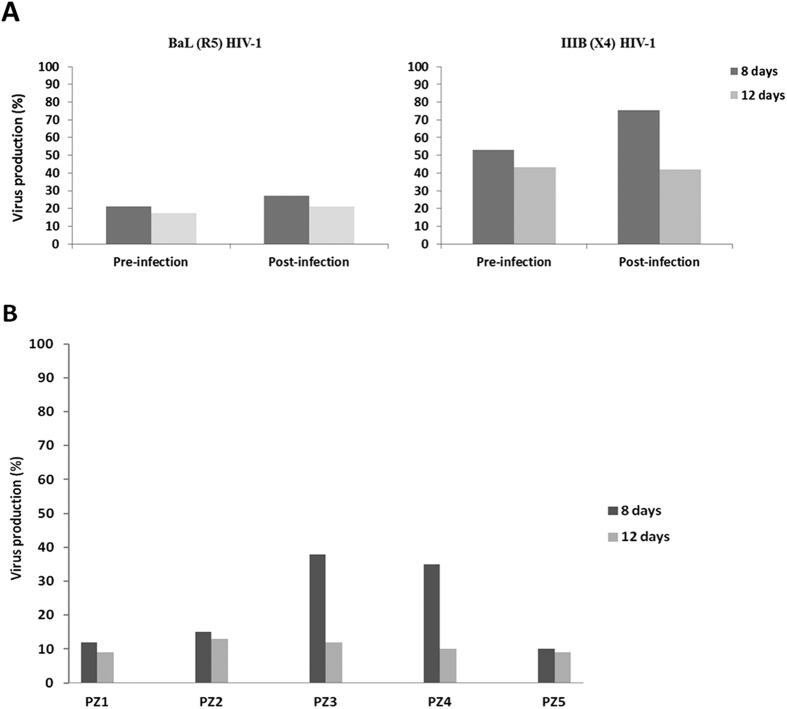
*In vitro* and *ex vivo* activity of K40H against HIV-1. (**A**) K40H (2 μM) was added to PBMCs cultures before (pre-) or after (post-) infection with BaL (R5, left panel) and IIIB (X4, right panel) HIV-1. (**B**) PBMCs from five HIV-1-infected patients (PZ1-5) were cultured in presence of K40H (2 μM). Virus production was assayed in the supernatants by detecting p24 HIV-1 antigen, on day 8 and 12 after infection. Ag production in untreated cultures corresponded to 100% of virus production. For all assay conditions, results are representative of mean values from 5 independent experiments, in all cases variability was less than 10%.

The *in vitro* antiviral activity of peptide K40H was evaluated by infecting peripheral blood mononuclear cells (PBMCs) from healthy donors with R5 (BaL) and X4 (IIIB) strains of HIV-1. The peptide (2 μM), added either before or after infection, was active against both R5 and X4 HIV-1. In fact, as shown in Fig. 2A, a significant decrease of p24 antigen production was observed in the supernatants of infected cultures at both day 8 and 12 post-infection. Interestingly, a more potent antiviral activity was seen against R5 strains, whose replication was inhibited by approximately 80% (at day 12). A highly significant difference (*p* < 0.001) between K40H-treated and untreated infected cells was always observed. Conversely, no significant decrease of p24 antigen production was observed in the supernatants of virus infected cultures on day 8 and 12 post-infection, when the irrelevant peptide S17K was added, even at the highest tested concentration (5 μM). Data obtained from the *ex vivo* assay confirmed the antiviral activity of peptide K40H (Fig. 2B).

Microscopic observation showed the complete absence of syncytium formation caused by the virus in K40H-treated infected cells in comparison to untreated infected cells.

#### Haemolytic, cytotoxic, and genotoxic effects

Peptide K40H was tested for haemolytic, cytotoxic and genotoxic effects on human erythrocytes, mammalian cells and PBMCs. No haemolytic activity was detected. Indeed, even at the highest tested concentration less than 1% of the erythrocytes lysed with reference to the negative control (0% lysis) consisting of erythrocytes suspended in phosphate buffered saline (PBS) in comparison to the positive control (erythrocytes suspended in PBS plus Triton 1%, 100% lysis). Peptide K40H was not cytotoxic when tested with LLC-MK2 cells as assessed by the use of resazurin as indicator in a cell viability assay. At the concentrations tested, mean absorbance values were not different for K40H-treated and untreated cells. No genotoxic activity was observed in the Comet assay performed on PBMCs. There were no significant changes in % tail DNA for PBMCs treated with 5, 10, and 20 μM K40H (0.26 ± 0.15, 0.23 ± 0.14, and 0.22 ± 0.04, respectively) in comparison with the value (0.23 ± 0.14) recorded for untreated PBMCs (negative control).

### *In vivo* toxicity and therapeutic activity of peptide K40H

Peptide K40H toxicity was preliminarily assessed in the *G. mellonella* model. Under the experimental conditions adopted, there was no significant difference in survival among untouched larvae, saline-injected, and peptide-injected larvae.

**Figure 3:**
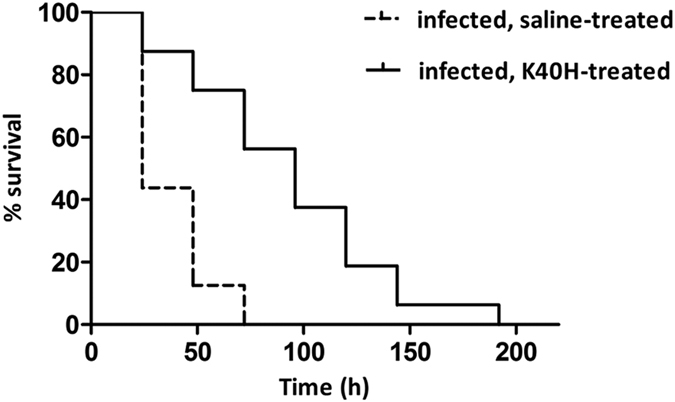
*In vivo* therapeutic activity of K40H. Effect of treatment with a single dose of peptide (10 μl from a 86.5 μM solution, 1 h postchallenge) on the survival of *Galleria mellonella* larvae (16/group) infected with 5 × 10^5^ *Candida albicans* cells. The survival curve of K40H-treated animals was significantly different (*p* < 0.0001) from that of control larvae as assessed by the log-rank (Mantel-Cox) test. Data reported are from one representative experiment out of two experiments with comparable results.

In two independent esperiments, a single dose administration of K40H led to a significant increase in the survival of *G. mellonella* larvae infected with *C. albicans* cells. There was a highly significant (*p* < 0.0001) statistical difference between the survival curves of treated and untreated larvae as assessed by the log-rank (Mantel-Cox) test. In Fig. 3 results from one representative experiment are shown. Median survival time was 24 h in saline-injected vs 96 h in K40H-treated group. At 72 h post-infection, 100% of the saline-injected larvae were dead, whereas more than 50% of the peptide-treated animals were still alive. Survival was prolonged up to 192 h in the treated group.

### Visualisation of the effects of peptide K40H on *C. albicans* cells by transmission and scanning electron microscopy

**Figure 4:**
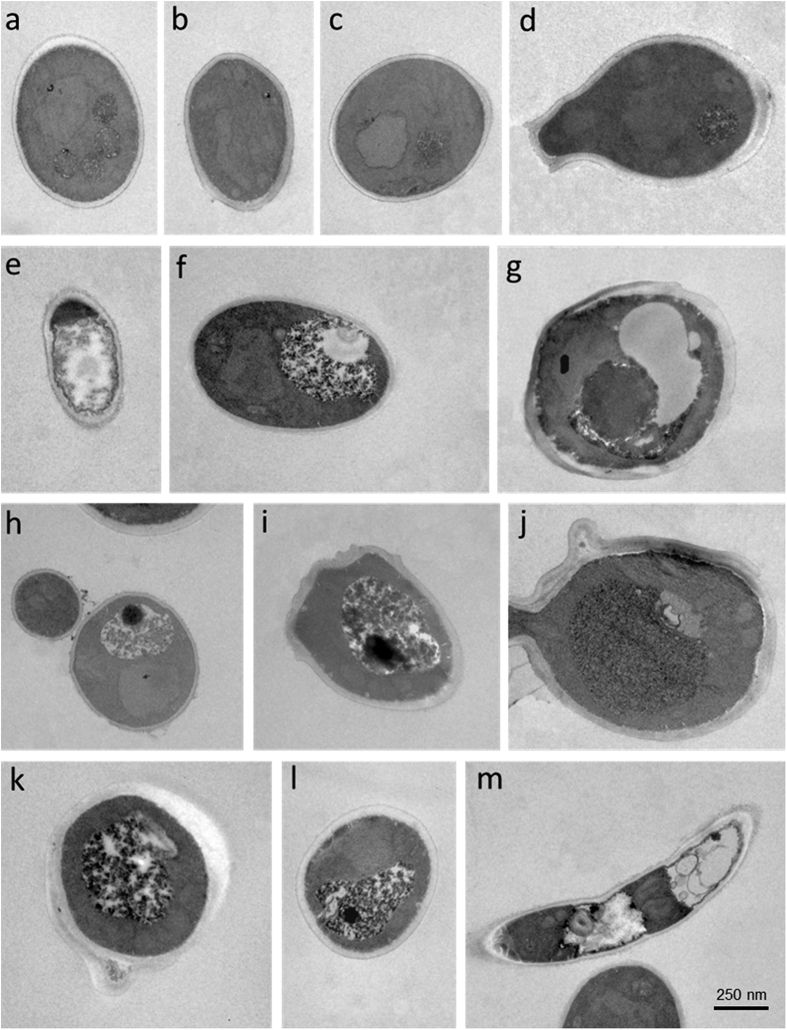
Transmission electron microscopy of *Candida albicans* cells treated with peptide K40H. Approximately 10^7^ yeast cells were incubated without (panels **a–d**) or with (panels **e–m**) K40H (32.5 μM) for 1 h. Treated cells presented membrane retraction and extensive cytoplasm disintegration (**e,m**), vacuolation (**g**), perinuclear and granular nuclear alterations (**f,h–l**), swelling and separation of an outer layer of the cell wall (**j,k**).

**Figure 5:**
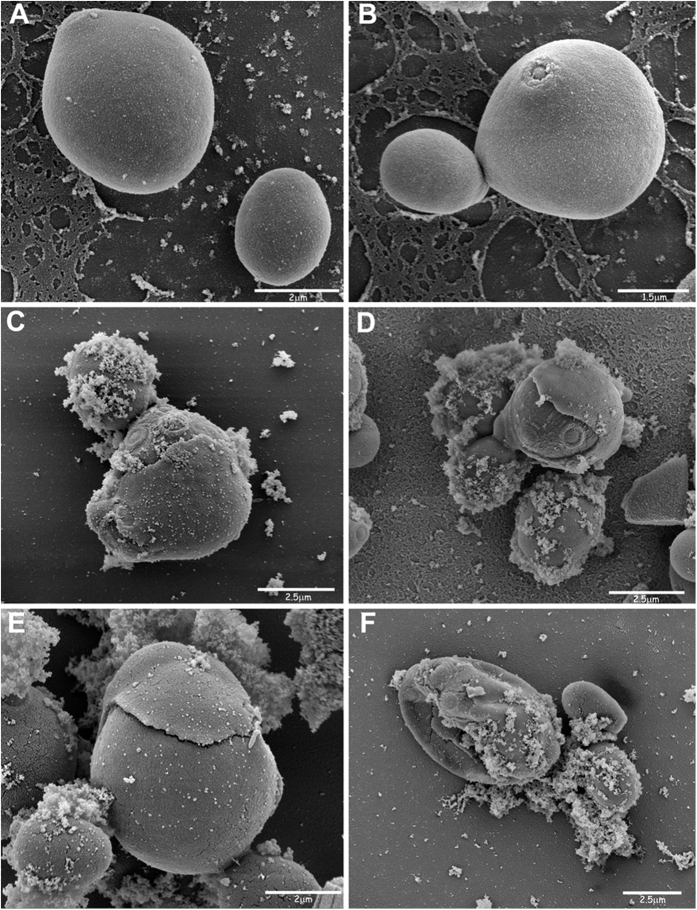
Scanning electron microscopy of *Candida albicans* cells treated with peptide K40H. Approximately 10^7^ yeast cells were incubated without (panels **A,B**) or with (panels **C–F**) K40H (130 μM) for 3 h. Treated cells presented a rugged surface and detachment of an outer layer of the yeast cell wall. Masses of cellular debris were seen.

As shown in Figs 4 and 5, treatment with peptide K40H caused gross alterations in the morphology of *C. albicans* cells in comparison to untreated controls. Cytoplasm disintegration and vacuolation were seen. Perinuclear and granular nuclear alterations were frequent. The cell wall in many yeast cells was swollen and the outer layer seemed to detach from the cell. Scanning electron microscopy showed masses of cellular debris; in some cells the cell wall presented a rugged surface. A remarkable effect was the apparent separation of an outer layer from the yeast cell wall.

### Binding of biotin-labeled peptide K40H to *C. albicans* cells

**Figure 6:**
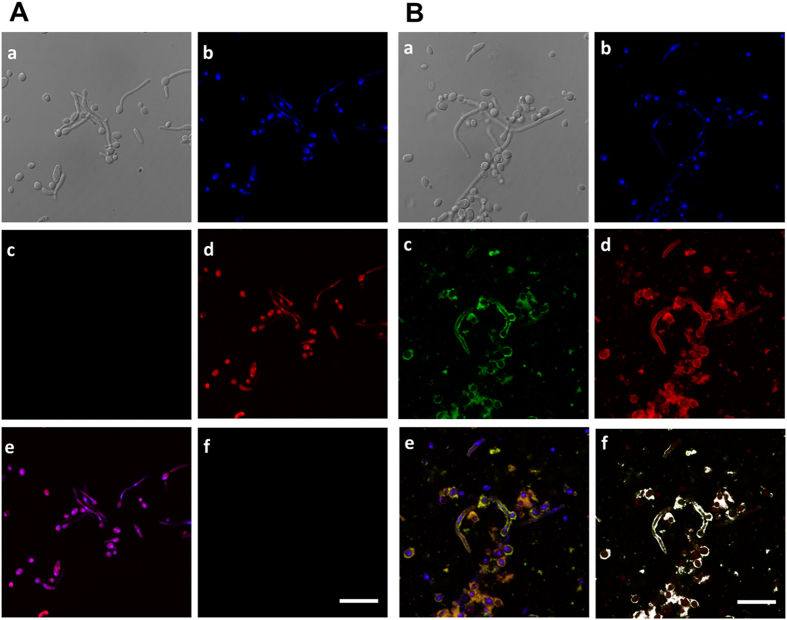
Binding of biotin-labeled K40H to F-actin on *Candida albicans*. Yeast cells were treated with biotin-labeled K40H (130 μM) for 3 h, fixed with formaldehyde and permeabilised with Triton X-100. Peptide-untreated cells (**A**) and peptide-treated cells (**B**) were stained with DAPI, streptavidin-FITC, and phalloidin-rodamine. Panel a: differential interference contrast microscopy; panel b: DAPI; panel c: streptavidin FITC; panel d: phalloidin-rhodamine; panel e: merge with superposition of blue, red and green fluorescence outputs (b–d) of the same cells; panel f: co-localisation points of c and d. Biotin-labeled peptide co-localises with F-actin. Scale bar, 20 μm.

Biotin-labeled peptide was used in order to evaluate binding to *C. albicans* cells. Confocal fluorescence microscopy showed that the peptide co-localises with phalloidin-rhodamine, a reagent that is quite specific for F-actin, mainly on cells that are undergoing germination (Fig. 6).

### Actin polymerisation caused by K40H

**Figure 7:**
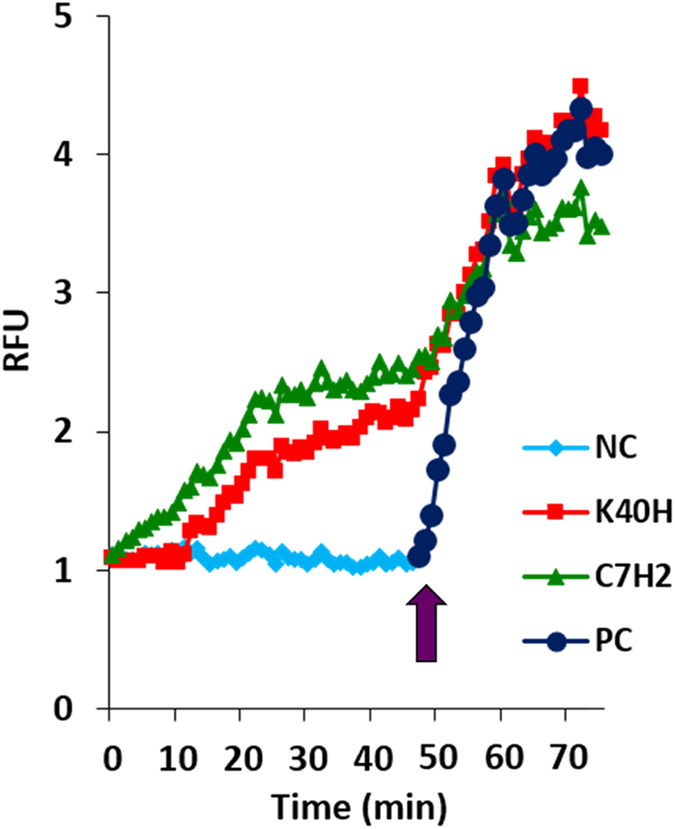
Actin polymerisation by K40H. Polymerisation of pyrene-labeled monomeric actin (G-actin) was assayed with K40H at 200 μM in comparison with a positively reacting peptide C7H2, in a time dependent kinetics. The fluorescence at 410 nm was measured (RFU, relative fluorescent units). At the end of 50 min of the stabilized pyrene-actin (NC, negative control), ATP-containing polymerisation buffer was added (arrow) and the steep polymerisation and intensity (PC, positive control) was compared to that obtained with the peptides.

Evidence was also obtained that peptide K40H binds to G-actin causing its polymerisation. Monomeric pyrene-labeled G-actin was incubated with K40H (200 μM), with a positive control represented by peptide C7H2 (an actin binding Ig CDR) and, after 50 min, with ATP-containing polymerisation buffer. In presence of K40H, polymerisation of G-actin was observed in a time-dependent manner in two steps of increasing intensity, similarly to that observed with C7H2 (Fig. 7).

## Discussion

We have previously demonstrated that synthetic peptides representing fragments of the constant regions of human Igs, selected through a database search according to pre-determined criteria, are endowed with antifungal activity^4^. Assuming that these Fc-peptides could be originated by cleavage of Abs *in vivo*, human sera were analysed by LC-ESI-HRMS/MS in the attempt to identify them. The search, however, gave negative results. The novelty on this work relies on demonstrating the presence in human serum of a phosphorylated peptide, K40H, undoubtedly derived from the constant region of human IgMs. The synthetic peptide K40H showed a potent activity against selected strains of pathogenic fungi and HIV-1 and exerted a therapeutic activity in *G. mellonella* larvae against systemic candidiasis. The results obtained in this model support the hypothesis of a possible posthumous activity of antibody-derived peptides *in vivo*.

Peptide K40H did not show toxic activity on mammalian cells *in vitro* nor in the *in vivo* model, as could be expected by a fragment of a physiological molecule naturally occurring in human serum.

Cytomorphological alterations seen by electron microscopy in *C. albicans* cells were compatible with the involvement of cytoskeleton with polarisation and perinuclear localisation. Confocal microscopy of treated *C. albicans* cells showed co-localisation of peptide K40H with phalloidin-rodamine indicating binding to F-actin. We also observed polymerisation of G-actin by K40H, which suggests an effect of the peptide on actin dynamics. Actin plays a multifunctional role coordinating the synthesis of the cell wall, cytoplasmic migration and organelle positioning, and its distribution correlates with the sites of wall expansion and exocytosis^7^.

A potent anti-HIV-1 activity especially against R5 HIV-1 strain was demonstrated. The molecular mechanisms by which the peptide K40H induces a strong inhibition of viral replication need further analysis to be fully understood. However, the findings supported the hypothesis that K40H may act during the first steps of infection by controlling the signalling pathways that are crucial for virus replication. Because studies of conformational and/or intermolecular interactions could exclude the involvement of K40H in the inhibition of co-receptor binding, viral regulatory proteins (TAT, REV and NEF) may be considered potential targets of the peptide on the basis of predicted electrostatic interactions. The peptide is phosphorylated in two close residues (Thr19 and Ser21), which increases the negative charge already present in this area, due to two aspartic acid residues located nearby. The strongly acidic region around the phosphorylated residues could constitute an attractive pole for an opposing basic region on a regulatory protein. The analysis of several structures of TAT and REV available from the PDB databank^8^ (PDB id-codes 1TIV, 2X7L, 2NEF, 4EN2) demonstrates in both proteins a basic domain, that corresponds to the nuclear localisation signal and RNA-binding region^9^,^10^. The NEF protein, instead, has a basic N-terminal sequence, able to bind cellular proteins and known to be required for inner plasma membrane targeting of NEF and virion incorporation^11^. The fact that these basic areas are so functionally relevant for the virus infectivity supports the hypothesis that they could be the target of K40H peptide, by means of an electrostatically driven interaction. The fact that a significant reduction of virus production was already evident when the cells were treated with the peptide before being infected, could not exclude a K40H effect on the target cell.

Other possible biological functions (antitumour, immunoregulatory), already recognised as characteristics of Ab-derived peptides, can be investigated. Mass spectrometry-based approaches may also be extensively used to search for other Ab-derived peptides in human sera from individuals in various clinical conditions.

Bioactive fragments of Abs, which are the mediators of humoral adaptive immunity, functionally recall peptides of the innate immunity. The finding of the anti-infective activity of a serum Ab fragment may shade the conventional distinction between adaptive and innate immunity and support the hypothesis that the immune system evolution pursued diverse mechanisms for host defense against infections.

A wide range of physiological proteins may contain functional peptides (cryptides or crypteins) able to exert biological activities often unpredictable and different from the parental proteins^12^,^13^,^14^. The existence of cryptides, hidden within a protein sequence, seems to be a common phenomenon and may reflect an evolutionary mechanism that can expand the range of biological activities associated with a particular molecule. As a matter of fact, Abs can be added to the list of proteins from which cryptides are derived. In previous decades, several studies have focused on biologically active peptides derived from Abs, most of which, including tuftsin, rigin, immunorphin, immunocortin and peptide p24, were shown to be involved in the regulation of the immune response^15^. Among these, particular interest was aroused by tuftsin, a tetrapeptide first described in 1970, a natural activator of phagocyte cells present in the CH2 domain of the heavy chain of immunoglobulins and released by the action of two specific enzymes^16^.

The isolation of K40H from human serum is a strong argument against the generally taught concept that all peptides when not promptly eliminated by renal filtration are short lived because of proteolytic degradation. It is clear here that certain peptides resist proteolysis and persist in the blood stream without being sequestered out as by protein chaperons or by cell interaction. If one looks for possible modifications of the native peptide, K40H is phosphorylated in two rather close threonine and serine residues separated by a hydrophobic amino acid. Regulation of proteolysis susceptibility is one mechanism whereby threonine/serine phosphorylation can regulate protein function^17^,^18^. Recently, the activity and resistance to hydrolysis of S6-phospho-bradykinin (pBk) could be associated not only with the bulky phosphate group but also with a significant flexibility reduction of the peptide in comparison with Bk, as observed by NMR analysis^19^. Phosphorylation is also a mechanism through which some intracellular peptides escape degradation and may act regulating protein interactions^20^.

The main site of phosphorylation of Ig fragments is still unknown, but macrophages and dendritic cells are choice candidates because they participate in the Ab-mediated clearance of soluble and particulate antigens. In the case of natural IgM molecules, several studies highlight their importance in opsonising microbes and dying immune cells for macrophage clearance^21^,^22^,^23^,^24^. IgM particularly promotes the clearance of small size particles^25^. Partially degraded IgM molecules in macrophages generate peptides that might induce and/or serve as substrates of kinases. Whereas peptide-induced activation of threonine/serine kinases in macrophages must be specifically demonstrated, a similar process has been described for tyrosine kinases. A peptide of 18 residues containing a cryptic domain from laminin-1, but not the intact protein, when incubated with macrophages stimulated tyrosine phosphorylation of several proteins including Erk1/2^26^ and the expression of uPa and MMP-9 proteases. As eminently secretory cells, macrophages release in abundance cytokines, phosphoproteins and most probably phosphopeptides that could escape degradation such as K40H.

Overall, the demonstration of the presence in human serum of an Ab-derived fragment endowed with direct fungicidal and antiviral activity could shed new light on the posthumous role that Abs may exert in anti-infective homeostasis. The innate immunity is the first line in host defense, generally lacking specificity and memory. It includes mechanical barriers, the mucociliary escalator, and anti-infective chemicals, proteins and peptides, mainly those of epithelial lining fluids. Lysozyme, secretory phospholipase A_2_, surfactant proteins, and broad-spectrum cationic, antimicrobial and antiviral peptides belonging to the histatin, cathelicidin and defensin families are present in high concentrations^27^. The next level of protection, catalysed by cytokines/chemokines, histamine, bradykinin and complement promote the influx of neutrophils to initiate local inflammation. Ab-derived peptides could add to this list of functional components of the innate immunity system. Degradation of Abs, especially those that had fluctuating titers as a function of infections or other antigenic stimuli, is a constant source of peptides that, if protected from further proteolysis or clearance as by phosphorylation, and achieving a suitable concentration in the bloodstream or in tissues, could act non-specifically, depending on their concentration, on targets such as bacteria, fungi, yeasts and viruses much like the cathelicidins and defensins do^28^,^29^,^30^. In our paper we described the K40H phosphorylated peptide, derived from IgM, which targets pathogenic yeasts and viruses and may represent, at least theoretically, an additional family of anti-infective peptides of the innate immunity system.

## Methods

### Ethics statement

Serum samples were collected from nine healthy subjects enrolled within the multi-centre project ELVIS (Evaluation of Long Term Non Progressors Viro-Immunological Study) at the Infectious Diseases Division, L. Sacco Hospital of Milan. Blood samples as a source of PBMCs were collected from five HIV-1-mono-infected (anti-retroviral therapy-naïve) viremic subjects and five healthy donors. All recruited individuals gave informed consent, in accordance with the Italian law and with the principles of the Declaration of Helsinki, after the approval of the Ethics Committee of the Istituto Scientifico Universitario San Raffaele, Milan, Italy, dated March 3, 2005.

Research did not involve interaction with the donors nor their identification.

### Identification of peptides derived from native antibodies in human sera through LC-ESI-HRMS/MS

#### Sample preparation

Serum samples (100 μl), filtered through 0.2-μm nylon filters and diluted with 100 μl of denaturing solution (7 M urea, 2 M thiourea and 20 mM dithiothreitol), were stirred at 4 °C for 1 h after addition of ice-cold acetone (9 ml). After centrifugation at 6000 *g* for 30 min at 25 °C, the precipitate was dissolved in 2 ml of a mixture of acetonitrile and 12 mM HCl (70/30%, v/v) and stirred at 4 °C for 1 h. The supernatant obtained after centrifugation was lyophilised and re-suspended in aqueous solution of formic acid 0.1% v/v before LC-ESI-HRMS/MS analysis. Serum samples were analysed in duplicate (injection volume 10 μl).

For identification purposes, standard solutions (100 nM) of synthetic Fc-peptides N10K and T11F^4^ were prepared in distilled water and analysed by LC-ESI-HRMS/MS. Serum samples were thus spiked after extraction with a standard mixture of N10K and T11F peptides at the final concentration of 10 nM before LC-HRMS/MS analysis to estimate the limits of detection of the method described below.

#### LC-ESI-HRMS/MS

Analyses were performed on a Surveyor LC system coupled online to a LTQ linear ion trap-Orbitrap XL mass spectrometer (ThermoFisher Scientific, San José, CA). Chromatographic separation was performed on a Kinetex C-18 silica-bonded stationary phase (100 × 2.0, 2.7 μm, 100 Å pore size) (Phenomenex, Torrance, CA) using a gradient solvent system [(A) aqueous formic acid 0.1% (v/v)/(B) 0.05% (v/v) formic acid in acetonitrile] as follows: 5% solvent B for 2 min, then a linear gradient from 5% to 60% B in 120 min at a flow rate of 200 ml min^−1^, and solvent B at 95% for 15 min to clean the column before re-equilibration. Mass spectrometric conditions were set as follows: electrospray voltage, 3.3 kV; 50 sheath and 8 auxiliary gas flow (arbitrary unit); capillary temperature, 275 °C; capillary voltage, 13 V; tube lens 85 V. The mass range covered by the instrument was configured to *m*/*z* 300–2000. The ion selection threshold for the LIT was set at 10^6^ with a maximum ion accumulation time of 200 ms. The most abundant ions were selected for data dependent acquisition MS/MS in the linear ion trap with the following settings: ion threshold, 5000; minimum intensity, 3000; maximum ion accumulation time, 25 ms; activation time, 10 ms, collision energy 30 eV. Dynamic exclusion (60 s) was utilised to minimise redundant selection of peptides for MS/MS.

#### Bioinformatic Analysis

Mass spectra were searched for against the UniProt human protein database using SEQUEST algorithm (Proteome Discoverer, ThermoScientific). A precursor mass tolerance of 10 ppm and a fragment ion tolerance of 0.6 Da and a maximum of two missed cleavages were allowed.

### Synthesis of serum Ab fragment K40H

The synthetic phosphopeptide (KSTKLTCLVTDLTTYDSVpTIpSWTRQNGEAVKTHTNISESH) K40H was prepared by solid phase peptide synthesis method using a multiple peptide synthesiser (*SyroII*, MultiSynTech GmbH) on a preloaded 2-chlorotrithyl resin (Novabiochem, Bad Soden, Germany). The fluoren-9-ylmethoxycarbonyl (Fmoc) strategy^31^ was used throughout the peptide chain assembly, utilising O-(7-Azabenzotriazol-1-yl)-N,N,N′,N′-tetramethyluronium hexafluorophosphate (HATU) as coupling reagent in the presence of N,N-diisopropylethylamine^32^. The side-chain protected amino acid building blocks used were: N-α-Fmoc-Nω-(2,2,4,6,7-pentamethyldihydrobenzofuran-5-sulfonyl)-L-arginine, N-α-Fmoc-γ-*tert*-butyl-L-glutamic acid, N-α-Fmoc-β-*tert*-butyl-L-aspartic acid, N-α-Fmoc-O-*tert*-butyl-L-tyrosine, N-α-Fmoc-O-*tert*-butyl-L-serine, N-α-Fmoc-O-benzyl-phospho-L-serine, N-α-Fmoc-O-benzyl-phospho-L-threonine, N-α-Fmoc-N-ε-(*tert*-butyloxycarbonyl)-L-lysine, N-α-Fmoc-N(im)-trityl-L-histidine, N-α-Fmoc-N-γ-trityl-L-glutamine, N-α-Fmoc-S-trityl-cystine, N-α-Fmoc-N-β-trityl-L-asparagine, and N-α-Fmoc-N(in)-(*tert*-butyloxycarbonyl)-L-tryptophan. After chain assembly, the peptide was deprotected and cleaved by trifluoroacetic acid in the presence of ethanedithiol 5% for 2.5 h, followed by precipitation with cold ether. Crude peptide was purified by a preparative reverse-phase C18 HPLC. Molecular mass of the peptide was confirmed by mass spectroscopy on a MALDI TOF-TOF mass spectrometer (model 4800 – Applied Biosystems). The purity of the peptide was in the 80–90% range as evaluated by analytical reverse phase HPLC.

### Evaluation of the *in vitro* microbicidal activity of peptide K40H

The microbicidal activity of the synthetic peptide K40H was evaluated *in vitro* by a previously described colony forming unit (CFU) assay against the selected fungal strains *C. albicans* SC5314, CA-6, SA40, AIDS68, and UM4, *C. glabrata* OMNI32, *C. neoformans* serotype A 6995, and *Malassezia furfur* 101^4^. Briefly, approximately 500 fungal cells were suspended in 100 μl of distilled water in the presence or absence (control growth) of the synthetic peptide K40H at scalar concentrations. In selected experiments, the irrelevant S17K peptide was used as a further control. After incubation at 37 °C for 6 h, fungal cell suspensions were plated on Sabouraud Dextrose agar (*C. albicans*, *C. glabrata*, *C. neoformans*) or on Sabouraud Dextrose agar added with 1% Tween 20 (*M. furfur*). The plates were then incubated at 30 °C and CFUs were enumerated after 48–72 h. Each assay was carried out in triplicate. The EC_50_ was calculated by nonlinear regression analysis using Graph Pad Prism 4.01 software, San Diego, CA, USA.

Time kinetics of K40H-mediated killing of *C. albicans* was determined by CFU assays after incubation of yeast cells for 30 min, 1, 2, 4 and 6 h with 4, 2 and 1 μM K40H.

### *In vitro* and *ex vivo* evaluation of peptide K40H activity against HIV-1

Assessment of *in vitro* antiviral activity of peptide K40H was performed following two different protocols, i.e. pre-infection treatment and post-infection treatment. PBMCs were purified from blood samples of healthy donors through Ficoll-Hypaque density gradient centrifugation according to the manufacturer’s protocol. In the first protocol, PBMCs were incubated with K40H (2 μM) for 2 h, then were washed and infected with BaL (R5) or IIIB (X4) HIV-1 strains at a multiplicity of infection of 0.5. After 4 h of adsorption, the cells were washed, suspended at 2 × 10^5^ cells/ml in RPMI 1640 medium supplemented with 10% fetal calf serum and 20 U/mL of rIL-2, and cultured in 96-well culture plates. Exogenous rIL-2 was added every 4 days. In the second protocol, PBMCs were infected as previously described and, after 4 h of adsorption, the cells were washed and K40H (2 μM) was added to the cultures. All assays were performed in duplicate.

Anti-HIV-1 activity of K40H was also evaluated through an *ex vivo* assay. PBMCs, isolated from R5 HIV-1-infected patients, were cultured in 96-well plates at the density of 1 × 10^6^cells/mL in RPMI 1640 medium with 10% fetal calf serum and 20 U/mL of rIL-2, which was re-added every 4 days. When the cells were seeded, the K40H peptide was added to the medium. All assays were performed in triplicate.

In both *in vivo* and *ex vivo* assays, on day 8 and 12 after infection the supernatants were harvested and tested for virus production by assaying p24 (HIV-1 p24 ELISA Ultrasensitive detection kit, Perkin Elmer); p24 Ag in the supernatants of untreated cultures corresponded to 100% of viral production. Mean values from five replicates were calculated.

In selected experiments, the irrelevant S17K peptide was also used as a control.

### Evaluation of the haemolytic, cytotoxic, and genotoxic activity of peptide K40H

The synthetic peptide K40H was tested for its haemolytic activity against human erythrocytes (blood group 0 Rh+) according to a previously described procedure^4^. Briefly, 25 and 50 μM K40H in PBS was added to erythrocyte suspensions in 300 μl (final erythrocyte concentration, 2.5% v/v). After 30 min and 2 h incubation at 37 °C, 100 μl of the samples was centrifuged at 800 *g* for 10 min. Release of haemoglobin was monitored by measuring the absorbance of the supernatant at 540 nm. Results were expressed as the percentage of lysed erythrocytes, where controls for zero haemolysis (blank) and 100% haemolysis consisted of erythrocytes suspended in PBS and Triton 1%, respectively.

Cytotoxicity against LLC-MK2 monkey kidney epithelial cells was determined by using resazurin, a redox potential indicator that is converted to fluorescent resorufin dye by metabolically active cells^33^. LLC-MK2 cells cultured in Minimal Essential Medium containing 10% fetal bovine serum were seeded in 96-well plates (5 × 10^5^ cells/ml, 100 μl/well) and incubated for 24 h at 37 °C in 5% CO_2_ atmosphere. The cells were then treated with 25 and 50 μM K40H in medium containing 2% serum for 24 h. Cells in medium without peptide served as control. After this period, cells were incubated with resazurin 44 μM in serum-free medium for 30 and 60 min at 37 °C, then fluorescence intensity was measured at 572 nm using a Wallac 1420 Victor plate reader (Perkin Elmer).

Genotoxic activity against human PBMCs was evaluated by alkaline Comet assay, according to a previously described procedure^4^. Briefly, PBMCs were treated for 2 h with different concentrations of K40H (5, 10, and 20 μM) at 37 °C in an atmosphere containing 5% CO_2_. Cells were exposed at 4 °C overnight to a lysis buffer (2.5 M NaCl, 10 mM Na_2_EDTA, 10 mM Tris-HCl, 1% Triton X-100 and 10% DMSO, pH 10). DNA unwinding was achieved over 20 min in an electrophoretic alkaline buffer (1 mM Na_2_EDTA, 300 mM NaOH, at 0 °C, pH > 13). The electrophoresis was carried out for 20 min (0.78 V/cm, 300 mA) at 0 °C in the same buffer, followed by neutralisation in 0.4 M Tris-HCl, pH 7.5. The slides, stained with 0.75 μl ethidium bromide (10 μg/ml) were examined with a fluorescent microscope (Leica DMLS), equipped with a BP 515–560 nm excitation filter and an LP 580 nm barrier filter and data were collected using an automatic image analysis system (Comet Assay III, Perceptive Instruments Ltd). Fifty randomly-selected cells per slide (two slides per sample) were analysed. DNA migration was evaluated by percentage of DNA in comet tail (Tail Intensity). Mean of Tail Intensity values is reported.

### Evaluation of *in vivo* toxicity and therapeutic activity of peptide K40H

To study *in vivo* toxicity and potential therapeutic efficacy of peptide K40H, the *Galleria mellonella* model was used^34^,^35^. Preliminarily, groups of sixteen larvae at their final instar stage (body weight 300 ± 30 mg) were inoculated directly into the haemocoel, via the last left pro-leg, with 10 μl of K40H solution (216 μM). Control groups consisted of larvae untouched or inoculated with 10 μl of saline solution. Larvae were then transferred into clean Petri dishes, one for each experimental group, incubated at 37 °C in the dark for 9 days, and scored daily for survival.

For experimental infection with *C. albicans* SC5314, larvae were inoculated with 10 μl of yeast suspension (5 × 10^5^ CFU/larva) directly into the haemocoel via the last left pro-leg. Two groups (16 larvae/group) were included: 1) yeast-inoculated followed by treatment with a single dose of peptide (10 μl from a 86.5 μM solution/larva) administered into a different proleg 1 h after yeast challenge; 2) yeast-inoculated followed by injection of sterile saline solution. Further controls included untouched larvae, larvae injected with saline solution alone to monitor the trauma, and yeast-inoculated larvae. Statistical significance of survival curve of animals was assessed by the log-rank (Mantel-Cox) test using Graph Pad Prism software.

### Transmission and scanning electron microscopy studies

For transmission electron microscopy studies, *C. albicans* ATCC 64548 cells (approximately 10^7^) were incubated in the absence (control) or presence of peptide K40H (325 μM) for 1 h, then treated as previously described^4^. Briefly, yeast cells were fixed with 2.5% glutaraldehyde in sodium cacodylate buffer, and were post-fixed in the same buffer with 1% osmium tetroxide for 2 h. After dehydration by ethanol gradient (70–100%), the pellet was embedded in Spurr resin. Semi-thin cuts (300 nm) were stained with 0.25% toluidine blue for observation in an optical microscope (Axiophot Zeiss, with plan achromat objective), followed by ultra-thin cuts (70 nm) which were contrasted with 0.5% uranyl acetate and 0.5% lead citrate and observed in an EM Jeol 100 CX transmission electron microscope.

For field emission-scanning electron microscopy, yeast cells, after incubation with peptide K40H (130 μM) for 3 h, were fixed in the glutaraldehyde-cacodylate buffer solution for 1 h at room temperature. After washing 4 times, twice for 10 min, once overnight and once for 10 min, with cacodylate buffer 0.1 M, the pellet was post-fixed in the same buffer with 1% osmium tetroxide for 1 h. The cells were washed 3 times, 10 min each with the same buffer, then were treated with 1% tannic acid in water for 30 min. After washing, twice for 5 min with water, the pellet was incubated with 1% osmium tetroxide for 30 min and washed 3 times for 5 min with water. Dehydration was carried out by immersion in ethanol in a 50% to 100% gradient (twice for 10 min at 50%; twice for 10 min at 70%; twice for 10 min at 90% and 3 times for 10 min at 100%). The pellet was dried with CO_2_, fixed on a support and gold coated in an ion-sputtering unit. The samples were observed in a FEI Quanta FEG 250 field emission-scanning electron microscope.

### Confocal microscopy studies

Confocal microscopy studies were carried out according to a procedure previously described^4^. In brief, 2 × 10^7^ *C. albicans* ATCC 64548 cells were incubated for 1 h at room temperature with biotinylated peptide K40H (130 μM), washed 3 times with PBS and fixed with 4% paraformaldehyde for 1 h. After permeabilisation in Triton X-100 cells were incubated for 1 h in the dark with streptavidin-fluorescein isothiocyanate (FITC), then washed and stained with phalloidin-rhodamine for 1 h, followed by DAPI staining for 1 h. Cells were washed 3 times with PBS and 40 μl of mounting medium was added to the pellet. A sample of 20 μl was mounted on coverslips and observed in a Confocal Leica SP5 microscope, with a 63 × 1.4 oil objective; the Z series was obtained in accordance with sampling criteria built in the software. Signal detection was obtained from DAPI, which stains the nucleus in blue, using excitation at 350 nm and emission at 470 nm; from streptavidin-FITC at 494 nm excitation and 520 nm emission, and from phalloidin-rhodamine, that stains F-actin in red, at 580 nm excitation and 604 nm emission.

### Actin polymerisation assay

Actin polymerisation was evaluated using the Actin Polymerization Biochem kit (Cytoskeleton, Denver, CO) following the manufacturer’s instructions and in accordance with a previously described procedure^36^. K40H or peptide C7H2 used as a positive control was added at 200 μM. Samples were read at 355 nm (excitation) and 410 nm (emission) in a fluorometer (SpectraMax-M2, Molecular Devices Software Pro 5.4, Sunnyvale, CA), using a kinetic mode. Pyrene-labeled monomeric G-actin was used as substrate. After 50 min, the actin polymerisation buffer (50 mM KCl, 2 mM MgCl_2_, 1 mM ATP) was added and the steep increase in the fluorescence compared with the polymerisation response obtained with the peptides.

## Additional Information

**How to cite this article**: Polonelli, L. *et al*. A Naturally Occurring Antibody Fragment Neutralizes Infectivity of Diverse Infectious Agents. *Sci. Rep*. **6**, 35018; doi: 10.1038/srep35018 (2016).

## Supplementary Material

Supplementary Information

## Figures and Tables

**Table 1:** *In vitro* fungicidal activity of K40H.

Yeast strains	EC_50_* (95% confidence intervals) [mol/liter] × 10^−6^
*Candida albicans* SC5314	0.64 (0.61–0.66)
*C. albicans* CA-6	1.98 (1.92–2.03)
*C. albicans* SA40	2.04 (1.96–2.12)
*C. albicans* AIDS68	1.21 (1.10–1.32)
*C. albicans* UM4	1.93 (1.86–2.00)
*C. glabrata* OMNI32	1.42 (1.14–1.77)
*Cryptococcus neoformans* 6995	0.87 (0.71–1.05)
*Malassezia furfur* 101	2.74 (2.53–2.96)

^*^EC_50_, half maximal effective concentration, calculated by nonlinear regression analysis using Graph Pad Prism 4.01 software.
