# Supplementary material for: A Naturally Occurring Antibody Fragment Neutralizes Infectivity of Diverse Infectious Agents
Source: Sci Rep. 2016 Oct 11;6:35018. doi: 10.1038/srep35018 (PMC5057116; doi:10.1038/srep35018)
Supplement: Supplementary Information [file srep35018-s1.doc]

**A Naturally Occurring Antibody Fragment Neutralizes Infectivity of Diverse Infectious Agents**

Luciano Polonelli, Tecla Ciociola, Lisa Elviri, Pier Paolo Zanello, Laura Giovati, Denise C. Arruda, Julián E. Muñoz, Renato A. Mortara, Giulia Morace, Elisa Borghi, Serena Galati, Oriano Marin, Claudio Casoli, Elisabetta Pilotti, Paola Ronzi, Luiz R. Travassos, Walter Magliani, Stefania Conti


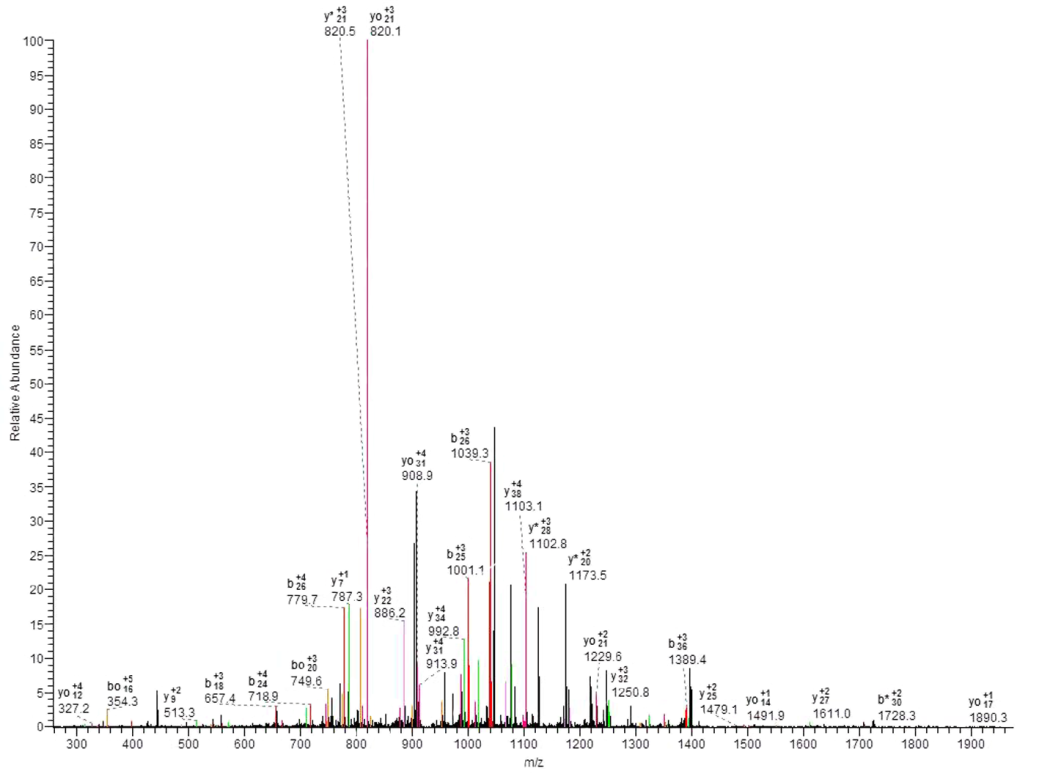


Supplementary Figure 1. LC-ESI-HRMS/MS mass spectrum of K40H IgM-derived peptide identified in human serum.
